# High-temperature supercapacitor with a proton-conducting metal pyrophosphate electrolyte

**DOI:** 10.1038/srep07903

**Published:** 2015-01-20

**Authors:** Takashi Hibino, Kazuyo Kobayashi, Masahiro Nagao, Shinji Kawasaki

**Affiliations:** 1 Graduate School of Environmental Studies, Nagoya University, Furo-cho, Chikusa-ku, Nagoya 464-8601, Japan; 2 Graduate School of Engineering, Nagoya Institute of Technology, Gokiso-cho, Showa-ku, Nagoya 466-8555, Japan

## Abstract

Expanding the range of supercapacitor operation to temperatures above 100°C is important because this would enable capacitors to operate under the severe conditions required for next-generation energy storage devices. In this study, we address this challenge by the fabrication of a solid-state supercapacitor with a proton-conducting Sn_0.95_Al_0.05_H_0.05_P_2_O_7_ (SAPO)-polytetrafluoroethylene (PTFE) composite electrolyte and a highly condensed H_3_PO_4_ electrode ionomer. At a temperature of 200°C, the SAPO-PTFE electrolyte exhibits a high proton conductivity of 0.02 S cm^−1^ and a wide withstanding voltage range of ±2 V. The H_3_PO_4_ ionomer also has good wettability with micropore-rich activated carbon, which realizes a capacitance of 210 F g^−1^ at 200°C. The resulting supercapacitor exhibits an energy density of 32 Wh kg^−1^ at 3 A g^−1^ and stable cyclability after 7000 cycles from room temperature to 150°C.

With increasing energy demands, global warming, and related environmental issues, considerable efforts have been devoted to the development of highly efficient and environmentally-friendly power sources. Supercapacitors, which operate by the charge and discharge of an electrical double layer (EDL), are becoming attractive power sources for industrial and domestic applications, and for renewable energy management, due to their higher energy density than conventional capacitors and higher power density than batteries^1^,^2^,^3^,^4^,^5^. In particular, the automobile industry has aggressively attempted to adopt this technology to improve fuel economy. A recent example is the application of supercapacitors to a regenerative braking energy system for vehicles^6^; when the vehicle decelerates, electricity generated by the alternator is transferred to the supercapacitor for storage. Although the details of this system are unknown, it is speculated that the supercapacitor is mounted on the coolest spot in the engine bay or the tire housing. However, the supercapacitor should ideally be integrated into the alternator to minimize energy losses associated with electricity transmission along wires. Note that the temperature of underhood automotive components, including the alternator, can reach *ca.* 150°C, although this is a worst-case scenario and not typical^7^.

The operation temperature of supercapacitors is generally determined by the thermal stability of the electrolyte. Both aqueous electrolytes (H_2_SO_4_ or KOH) and organic electrolytes (propylene carbonate or acetonitrile) limit the operation temperature to typically 100°C or lower, due to their low boiling points^8^,^9^,^10^. In contrast to these electrolytes, room-temperature ionic liquids (RTILs) are stable even at 300°C, due to the absence of solvent^11^; however, the ionic conductivity of these materials at room temperature is as low as a few millisiemens per centimeter^12^, which results in low power densities. Moreover, it is necessary to use a special clay separator when RTIL-based supercapacitors are operated at elevated temperatures^13^. (Separators such as cellulose papers, polymers, and glass wool shrink under such conditions, causing electrical shorts in the device.) For next-generation automotive applications, high ionic conductivity and stability of the electrolyte over a wide temperature range (−30 to preferably ≤200°C) will be crucial for the operation of high-temperature supercapacitors.

We have recently reported that the proton conductivity of metal pyrophosphates (Sn(IV)P_2_O_7_ or Fe(III)_0.5_Ta(V)_0.5_P_2_O_7_) could be increased to *ca.* 10^−1^ S cm^−1^ between 100 and 300°C by the partial substitution of Sn^4+^ cations with low valency cations, such as In^3+^ and Al^3+^, or by the introduction of a Fe^3+^ deficiency into the bulk^14^,^15^,^16^, which meets this criterion to realize practical application at high temperatures. It should also be noted that the observed high proton conductivity is almost independent of *P*H_2_O in the atmosphere, which is a useful characteristic in a closed system such as a supercapacitor. Furthermore, these compounds are anhydrous, which allows the use of charge voltages above 1 V for the supercapacitor. An additional benefit of these compounds is that a dense and flexible electrolyte membrane can easily be synthesized by binding the electrolyte powder with a small quantity of polytetrafluoroethylene (PTFE). Different types of solid electrolytes have been reported in the literature on supercapacitors, such as gel-form electrolytes composed of polyvinyl alcohol (PVA) and H_2_SO_4_^17^ or H_3_PO_4_^18^ and a Nafion polymer electrolyte^19^. However, the high-temperature performance of EDL-type supercapacitors with these electrolytes has not been studied thoroughly.

In spite of these efforts, the capacitance of so-called solid-state supercapacitors still remains strongly dependent on the protonics of the electrode employed because the EDL is formed only at the interface of the electrolyte and electrode. Unlike liquid electrolytes, the portion of the electrode away from the interface cannot form a contact with the solid electrolyte. One promising method to address this is to homogenously distribute an ionomer over the electrode layer, which provides a number of accessible sites for the electrolyte ions^19^. Accordingly, the electrochemical and physicochemical properties of the electrode ionomer are also very important factors that determine the supercapacitor performance at high temperatures. The goals of the present work were to (1) optimize the electrolyte and ionomer materials, (2) improve the surface utilization of micro- or mesoporous carbon, and (3) conduct capacitor tests using an optimized combination of electrolyte, ionomer, and carbon from room temperature to 200°C.

## Results

### Electrolyte and ionomer materials

**Figure 1:**
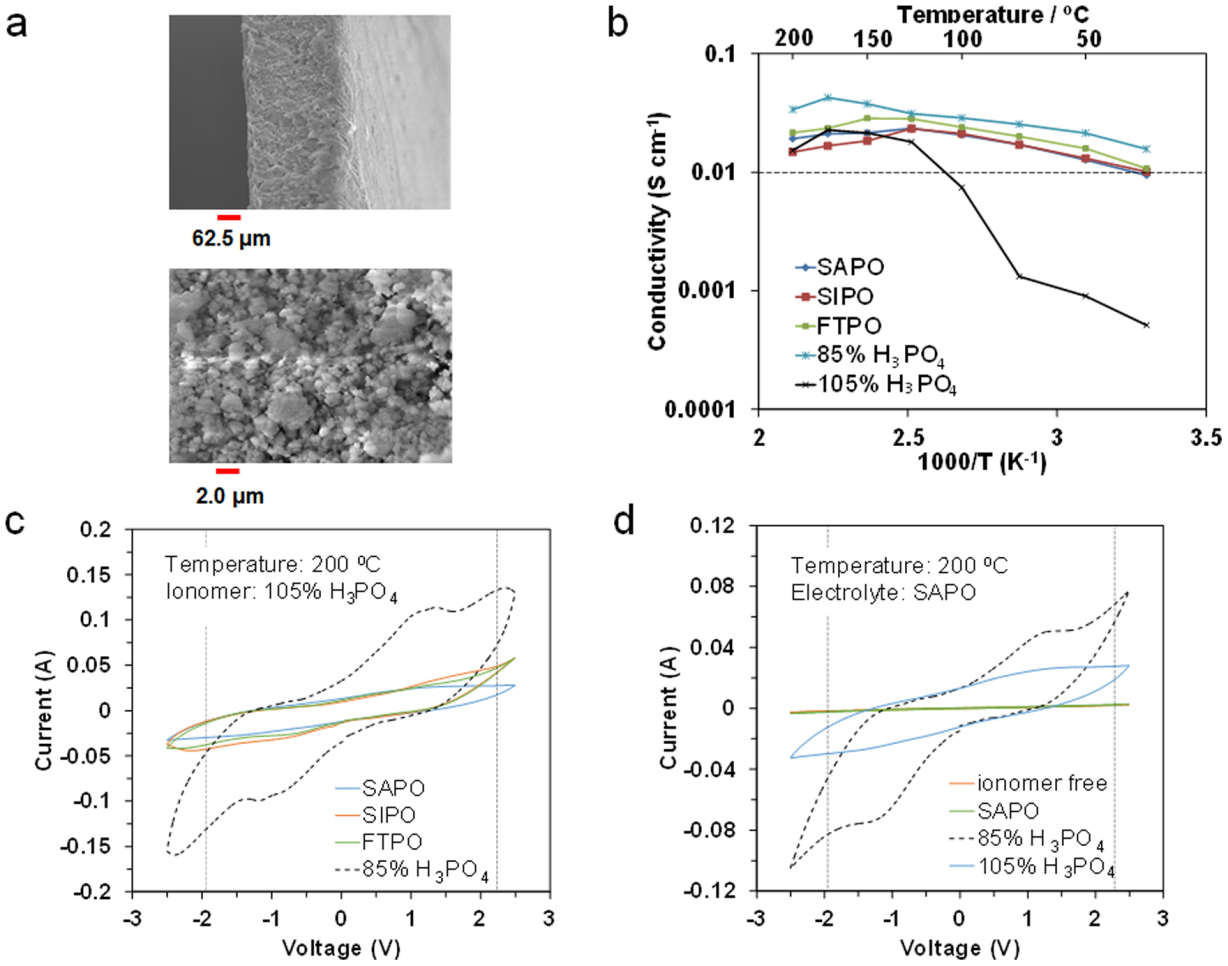
Characterization of electrolyte and electrode ionomer materials. a) SEM images of the SIPO-PTFE composite membrane. b) Temperature dependence of proton conductivity for the SIPO, SAPO, and FTPO-PTFE composite membranes, and 85% and 105% H_3_PO_4_. c) CV profiles for the capacitors with the SIPO, SAPO, and FTPO-PTFE composite membranes, and 85% H_3_PO_4_ electrolyte at 200°C, where 105% H_3_PO_4_ was used as the electrode ionomer. d) CV profiles for the capacitors with the SAPO, 85% and 105% H_3_PO_4_ ionomers at 200°C, where the SAPO-PTFE composite membrane was used as the electrolyte.

The morphology of a Sn_0.9_In_0.1_H_0.1_P_2_O_7_ (SIPO) composite membrane, as an example, was evaluated by scanning electron microscopy (SEM) analysis (Fig. 1a). Homogeneity ranging from submicrometers to a few micrometers was established. Similar membrane samples fabricated with the same technique could be produced with thicknesses of 75 to 500 μm. The gas permeation of the composite membranes with various thicknesses was evaluated at 150°C to define the critical limit of thickness (Supplementary Information, Fig. S1). No gas penetration was observed for membranes with thicknesses above 200 μm, which indicates that open pores are absent in the membrane sample. Thus, subsequent experiments were conducted using 250 μm thick membranes. The ionic conductivity for the SIPO, Sn_0.95_Al_0.05_H_0.05_P_2_O_7_ (SAPO), and Fe_0.4_Ta_0.5_H_0.3_P_2_O_7_ (FTPO) composite membranes was compared with those for 85 and 105% H_3_PO_4_ in the temperature range from room temperature to 200°C (Fig. 1b). The results for the composite membranes revealed two common features. Firstly, no abrupt decrease in the ionic conductivity with temperature was observed in the temperature range tested, which is similar to the case for H_3_PO_4_. The temperature where the maximum electrical conductivity occurred was approximately 150°C, which is not achieved for any other conventional aqueous or organic ionic conductors. Secondly, all the tested samples exhibited high ionic conductivities of 0.01 S cm^−1^ or higher, even at room temperature, which indicates the possibility that the three composite membranes, as well as 85% H_3_PO_4_, can function as both electrolyte and ionomer materials. However, 105% H_3_PO_4_ is unsuitable as an electrolyte, due to its low ionic conductivity below 100°C, although it may be used as an ionomer. (Ion transport in the long-distance region is not required for the ionomer.) Cyclic voltammetry (CV) profiles for the capacitors were measured using the three composite membranes as electrolytes in the voltage range from −2.5 to +2.5 V with a scan rate of 10 mV S^−1^ at 200°C (Fig. 1c; data for the capacitor with the 85% H_3_PO_4_ electrolyte is included). The composite membranes exhibited more rectangular shaped profiles than 85% H_3_PO_4_. This is attributed to the lower current in the composite membranes, which reduces the influence of the ohmic resistance on the CV characteristics. Another explanation is the appearance of two broad peaks around −1 and +1 V in the CV profile for 85% H_3_PO_4_, which indicates that faradaic reactions based on the decomposition of water molecules occur in the capacitor. More importantly, the composite membranes, especially SAPO, successfully exhibited capacitive behavior up to a ±2 V voltage range without any electrical short-circuit. CV profile measurements of capacitors with various proton conductors as the ionomer were also performed at 200°C (Fig. 1d). However, mixing the carbon powder with the SAPO powder was not effective for the formation of an EDL on the carbon surface. The 85% H_3_PO_4_ ionomer caused the decomposition of water molecules in the ionomer, similar to the case when used as an electrolyte. In contrast, the 105% H_3_PO_4_ ionomer did not exhibit such faradaic behavior in the ±2 V voltage range. It should be noted that these results show the withstanding voltage properties for each cell component during the limited CV cycles. SEM images before and after immersion of the electrode into 105% H_3_PO_4_ show that the carbon particles are homogeneously and intimately covered with 105% H_3_PO_4_ (Supplementary Information, Fig. S2), which suggests that the wettability between the two media is good, at least for the external surface of carbon particles. Therefore, the optimal electrolyte and ionomer for the present capacitor were determined to be SAPO and 105% H_3_PO_4_, respectively.

### Modification of carbon

**Figure 2:**
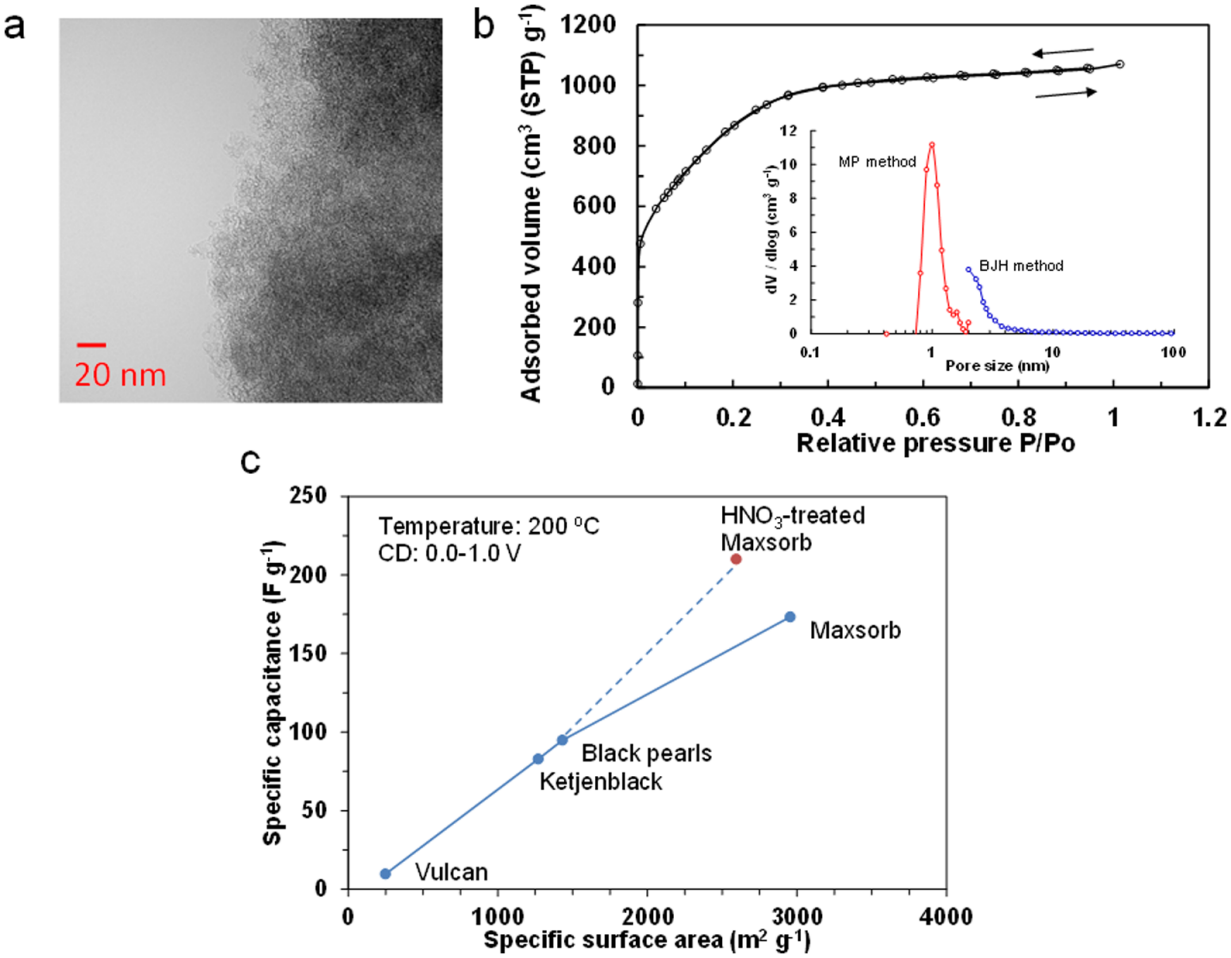
Characterization of carbon electrode materials. a) TEM image of the Maxsorb carbon. b) N_2_ adsorption/desorption isotherm for the Maxsorb carbon at 77 K. The inset shows the pore size distribution. c) Relationship between capacitance and BET specific surface area. The capacitance was estimated from the discharge curve measured with a current density of 3.6 A g^−1^ at 200°C.

The microstructure of pure Maxsorb was observed by transmission electron microscopy (TEM) analysis (Fig. 2a). The framework of Maxsorb is a poorly defined porous structure rather than a wormhole-like porous structure. It was difficult to estimate the pore size distribution from the micrograph; however, both micro- and mesopores were likely present. The extent of the porosity was further investigated by N_2_ adsorption-desorption isotherm measurements (Fig. 2b). Maxsorb has a type I isotherm, which is characteristic of mainly microporous materials^20^. The micropore and mesopore volumes calculated by the standard micropore (MP) and Barrett-Joyner-Halenda (BJH) methods were 1.60 and 0.87 cm^3^ g^−1^, respectively. The Brunauer–Emmett–Teller (BET) surface area was 2955 m^2^ g^−1^, which is considerably larger than those for the other activated carbons (Vulcan, 248 m^2^ g^−1^; Ketjen Black, 1270 m^2^ g^−1^; Black Pearls, 1431 m^2^ g^−1^).

To evaluate the electrochemical properties of the Maxsorb carbon sample, a galvanostatic charge-discharge profile was measured with a current density of 3.6 A g^−1^ at 200°C (Supplementary Information, Fig. 3S). The capacitance (C) was calculated according to^21^,^22^:
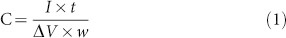
where *I* is the discharge current, *t* is the discharge time, *ΔV* is the voltage difference of discharge, and *w* is the total mass of the two carbon electrodes. The result was compared with the capacitances measured for Vulcan, Ketjen Black, and Black Pearls under the same conditions (Fig. 2c). The Maxsorb carbon had the highest capacitance among the tested carbon samples, although it was somewhat lower than that expected from its BET surface area when assuming that the capacitance is proportional to the surface area. This deviation can be explained by the restricted diffusion of ionomer ions in the narrow micropores of Maxsorb, which reduces surface utilization for the EDL^23^,^24^; all of the micropore surfaces in this sample were not wetted by the ionomer. It has been reported that functional groups can enhance the wettability of carbon surfaces and consequently increase the capacitance of the carbon^25^,^26^; therefore, the Maxsorb sample was treated with HNO_3_ at room temperature to form acidic surface oxides. Table 1 summarizes the physicochemical properties of the Maxsorb carbon sample before and after the HNO_3_ treatment. The weight of the treated sample was increased to 108% of the initial value after HNO_3_ treatment. An increase in the O/C atomic ratio from 0.044 to 0.066 was also determined by X-ray photoelectron spectroscopy (XPS) analysis (Supplementary Information, Fig. S4). While the BET surface area of Maxsorb after HNO_3_ treatment was somewhat lower than that before treatment, there was no difference in the intensity ratio of the D-band (*ca*. 1340 cm^−1^) to the G-band (*ca*. 1580 cm^−1^) in the Raman spectra of the two samples (Supplementary Information, Fig. S5). Therefore, it is confirmed that the carbon surface is partially oxygenated without significant structural deformation. As a result of this modification, the capacitance of the sample was enhanced to 210 F g^−1^ (Fig. 2c), where a good relationship between the capacitance and surface area was established.

### Capacitor performance from room temperature to 200°C

**Figure 3:**
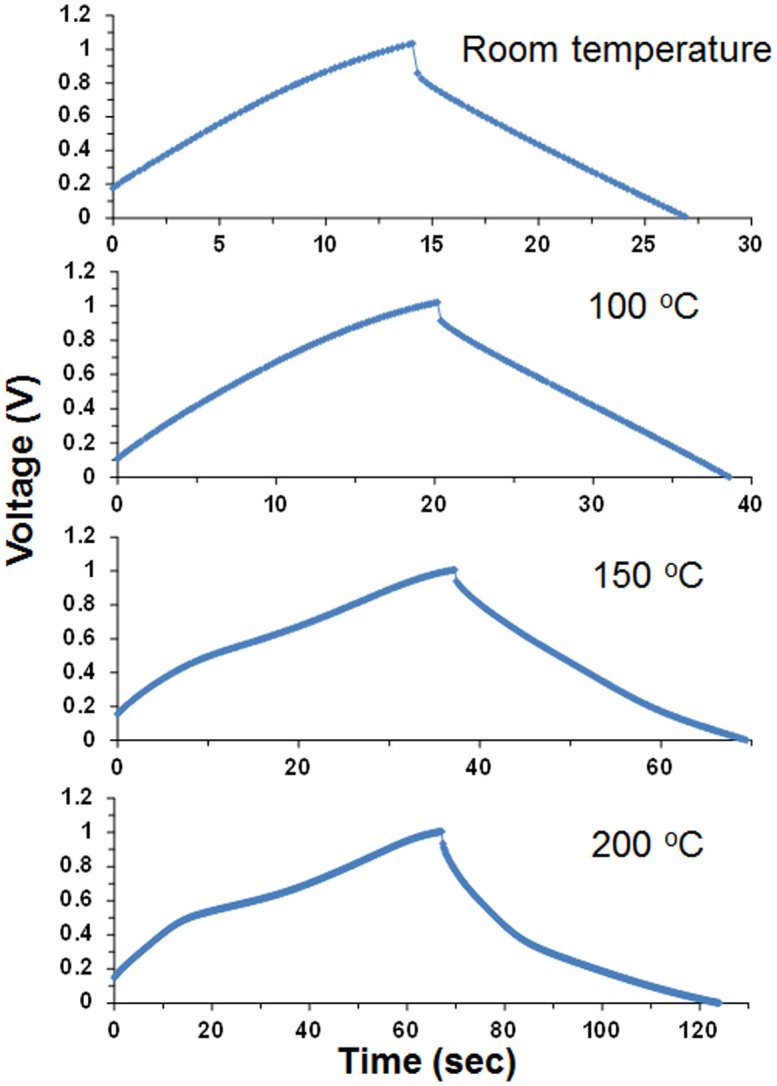
Galvanostatic charge-discharge curves for capacitor with SAPO-PTFE composite electrolyte, 105% H_3_PO_4_ electrode ionomer, and HNO_3_-activated carbon measured at various temperatures, where the current density was set at 4.5 A g^−1^.

Galvanostatic charge-discharge profiles for the capacitor with the optimized cell components were measured at different temperatures (Fig. 3). The charge/discharge time at 4.5 A g^−1^ increased significantly with the operation temperature, but the voltage curve became more nonlinear. The increase in the charge/discharge time is mainly due to a decrease in the internal resistance of the capacitor (Supplementary Information, Fig. S6). At low temperatures, electrochemical impedance spectroscopy (EIS) spectra showed higher ohmic resistance in the high-frequency range and a larger semicircular arc in the mid-frequency range. The former is related to the ionic resistance of the electrolyte and ionomer materials, the electrical resistance of the carbon material, and the contact resistance at the interface of the carbon material and current collector, many of which are improved by an increase of temperature. The latter is associated with the pore structure of the carbon material^27^. The micropore volume for Maxsorb is larger than the mesopore volume, which leads to a decrease in ionomer ion adsorption on the carbon surface. However, this would be enhanced with an increase of temperature, because ion transport is accelerated under such conditions. A similar explanation is also applicable to the high capacitance observed for Maxsorb at 200°C (Fig. 2c). On the other hand, the nonlinearity in the voltage curve is attributed to redox reactions on the carbon surface with functional groups such as carboxyl, carbonyl, and phenolic species. A pseudocapacitance is developed simultaneously with the double-layer capacity, which causes a distorted voltage curve during the charge-discharge processes^25^,^26^. This interpretation is supported by the EIS spectra for the capacitor (Fig. S6). Nyquist plots measured at all temperatures except 200°C showed ideal capacitive behavior with near vertical lines parallel to the imaginary axis at low frequencies. However, a slight deviation from the theoretical 90° vertical line was observed for the capacitor at 200°C, which indicates that the carbon electrodes do not function as planar electrodes under such conditions^28^.

**Figure 4:**
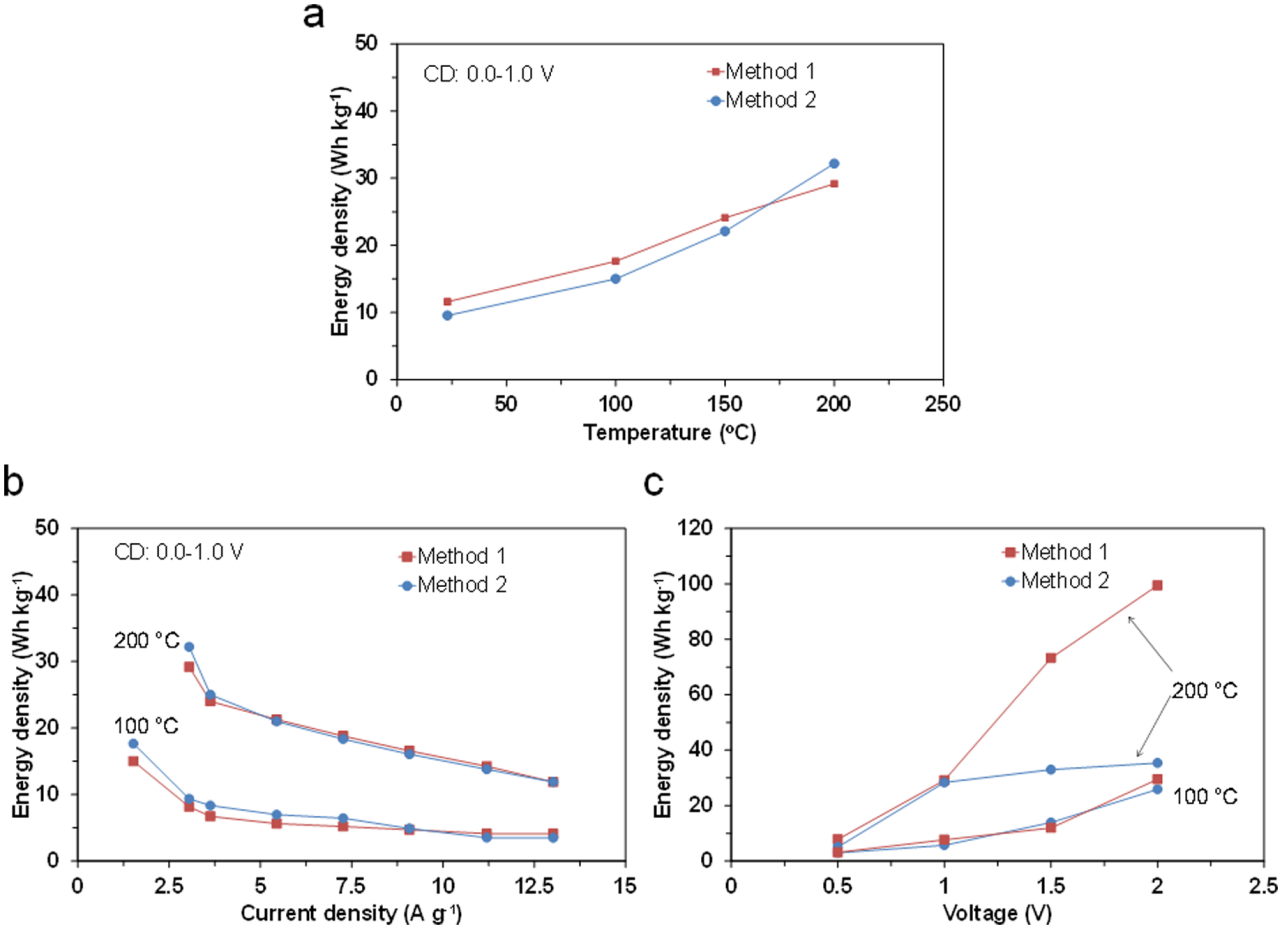
Electrochemical performance of capacitor with SAPO-PTFE composite electrolyte, 105% H_3_PO_4_ electrode ionomer, and HNO_3_-activated carbon. a) Energy density as a function of temperature, where the current density and the charge voltage were set in the range of 1.5–3 A g^−1^ and at 1 V, respectively. b) Energy density as a function of current density at 100 and 200°C, where the charge voltage was set at 1 V. c) Energy density as a function of charge voltage at 100 and 200°C, where the current density was set in the range of 3–6 A g^−1^. The procedures for Methods 1 and 2 are described in the manuscript.

The energy density (E) was calculated from the charge-discharge curve according to Eq. 2 (Method 1)^21^,^22^:
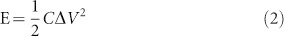
where *C* is the capacitance normalized according to the total mass of the two carbon electrodes, and Δ*V* is the voltage change (excluding the IR drop) with discharge time. The energy density (E) was also calculated by integrating the area under the voltage-time curve during discharge, multiplying by the current, and then dividing by the total mass of the two carbon electrodes (Method 2), because some of the charge-discharge curves are not typically triangular in shape. As reflected by the charge-discharge profiles (Fig. 3), the energy densities estimated by Methods 1 and 2 were both strongly dependent on the operation temperature (Fig. 4a). In particular, high energy densities of 29 and 32 Wh kg^−1^ were achieved at 200°C using Methods 1 and 2, respectively. The effects of the current density and charge voltage on the energy density were also measured at 100 and 200°C. An increase in the current density had a negative effect on the performance at both temperatures; however, the energy density obtained at 200°C maintained a moderate value of 12 Wh kg^−1^ for Methods 1 and 2, even at a very high current density of 13 A g^−1^ (Fig. 4b). In contrast, an increase in the charge voltage had a positive effect on the performance in a different manner at the two temperatures (Fig. 4c). The energy densities estimated using Methods 1 and 2 were almost comparable in the voltage range from 0 to 2 V at 100°C, whereas the energy density estimated from Method 2 was much lower than that estimated with Method 1, especially at voltages above 1 V at 200°C, which implies that this capacitor exhibits different voltage windows, depending on the operation temperature, and this point will be discussed later.

## Discussion

**Figure 5:**
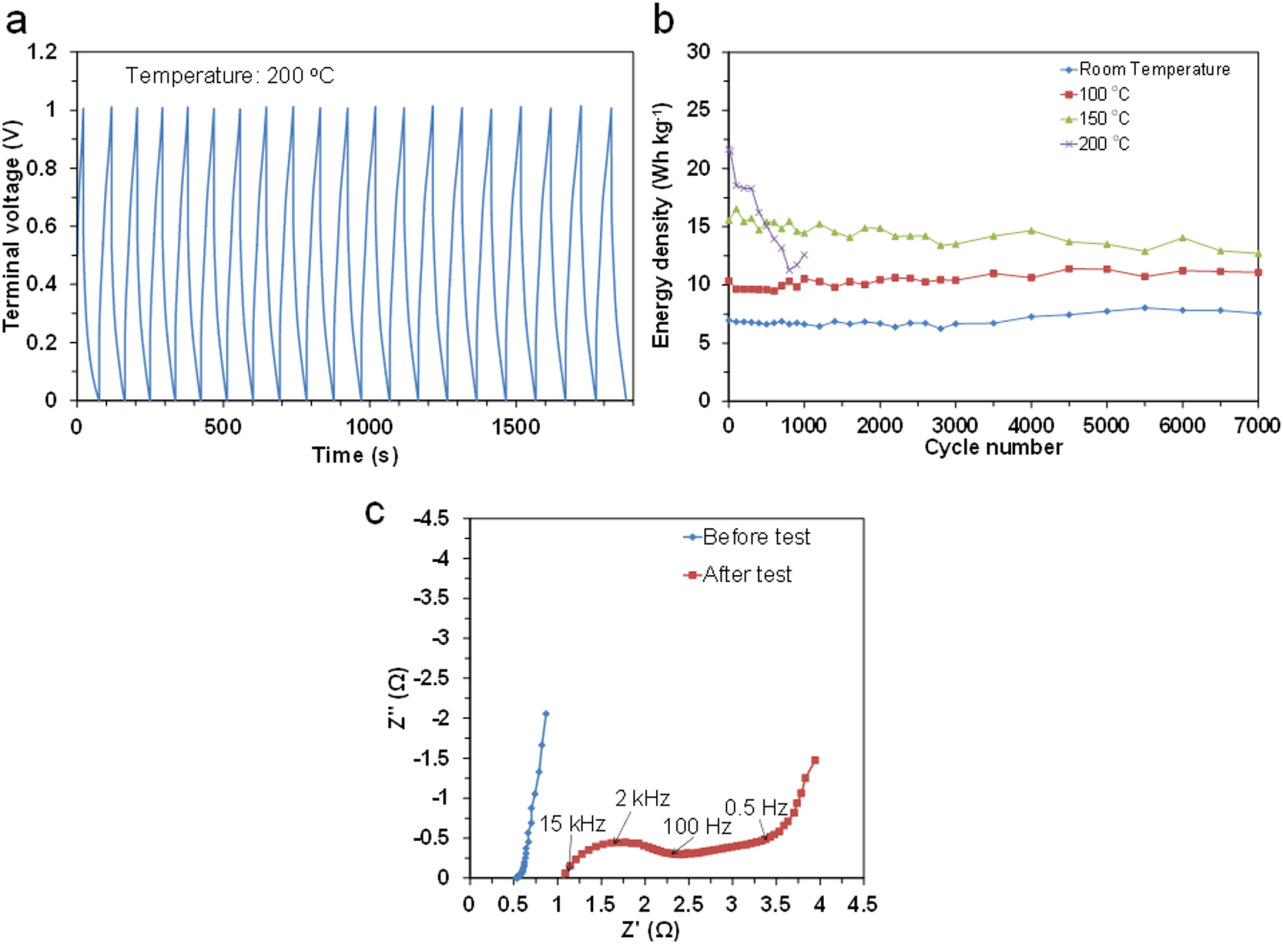
Cyclability of capacitor with SAPO-PTFE composite electrolyte, 105% H_3_PO_4_ electrode ionomer, and HNO_3_-activated carbon. a) Galvanostatic charge-discharge curve measured with a current density of 6 A g^−1^ at 200°C. b) Energy density as a function of cycle number measured at various temperatures with the current density set in the range of 4.5–6 A g^−1^. c) EIS spectra for the capacitor before and after 1000 cycles at 200°C.

The limitations of the present capacitor and directions for future research are discussed here. The cyclability of the capacitor between room temperature and 200°C was evaluated by galvanostatic charge-discharge measurements at various current densities from 4.5 A g^−1^ (room temperature) to 6 A g^−1^ (200°C), where the coulombic efficiency was adjusted to above 90%. In all tests, overshoots over 1 V upon charge or under 0 V upon discharge were scarcely observed (see for instance Fig. 5a). The capacitor showed stable cyclability at all the tested temperatures, except 200°C (Fig. 5b). At room temperature and 100°C, the energy density was either unchanged or very slightly increased with the cycle number. At 150°C, the energy density remained at 83% of the initial value after 7000 cycles. In contrast, at 200°C, the energy density continuously decreased as the cycle number increased. In addition, the coulombic efficiency was reduced to 72% after 1000 cycles. There are two reasons for this abrupt deterioration. One is a decrease in the adhesion of the sealing tape with time at 200°C, which allowed the incorporation of air into the capacitor. Thus, it is necessary to improve the sealing of the device. Another is an increase in the internal resistance of the capacitor (Fig. 5c), where both the electrochemical charge region (100 Hz–1.5 kHz) and the diffusion region (0.5–100 Hz) are more enlarged than the ohmic region after the cycle test. The former two impedances are determined mainly by the motility, mobility, and distribution of the ionomer in the micropores of the carbon electrode^29^, which suggests that the ionomer or the carbon surface deteriorate during the cycle test at 200°C.

**Figure 6:**
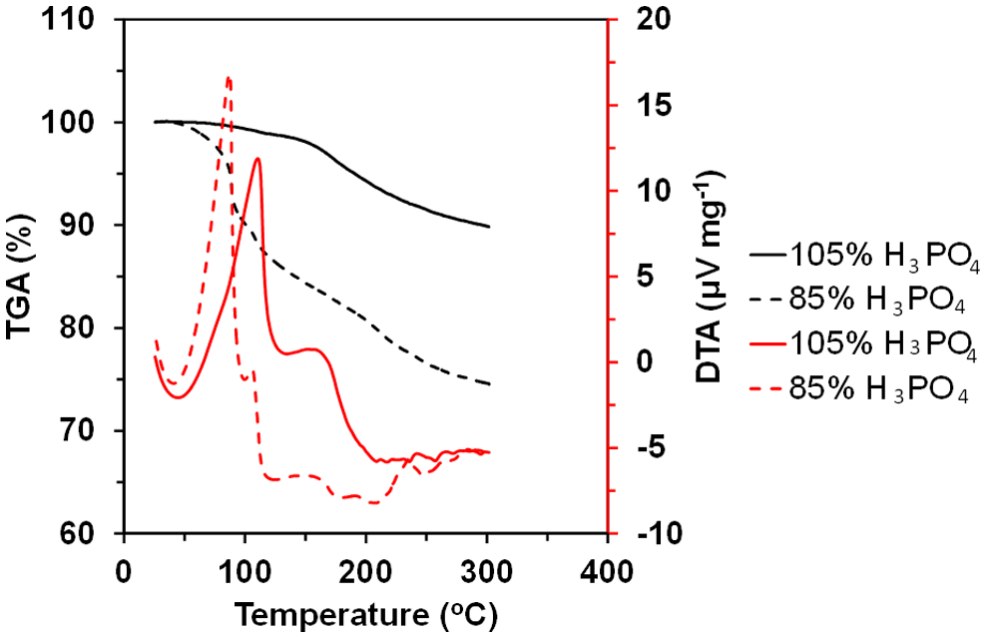
TG-DTA curves for 85% and 105% H_3_PO_4_ measured in a N_2_ flow.

The behavior of 105% H_3_PO_4_ at elevated temperatures was compared with that of 85% H_3_PO_4_ by thermal analysis (Fig. 6). The thermogravimetric analysis (TGA) curves for 105% and 85% H_3_PO_4_ indicated weight losses of 10% and 25%, respectively, between 50 and 300°C, which are commonly accompanied by endothermic peaks in the differential thermal analysis (DTA) curves. The endothermic peaks observed above 100°C can be ascribed to the dehydration of H_3_PO_4_ to form pyrophosphoric, triphosphoric, or polyphosphoric acid, because a gel-like solid was present as a residue in the aluminum pan after the measurements. More attention should be given to the dehydration of 105% H_3_PO_4_ above 150°C, which is almost consistent with the deterioration onset temperature of the capacitor. Thus, the dehydrative condensation of the ionomer is responsible for the poor cyclability at 200°C. Furthermore, the narrow voltage window between 0 and 1.0 V at 200°C (Method 2 in Fig. 4c) could also be due to the decomposition of water molecules produced by dehydration of the ionomer, which causes a rapid decrease in the cell voltage from 1.5 to ca. 1.0 V over time during discharge (Supplementary Information, Fig. S7). (It should be noted that the energy density estimated by Method 1 could not reflect the rapid voltage drop, because the capacitance (C) in Eq. (2) is calculated from the slope of the discharge curve between 0 and 1.0 V.) The instability problems at 200°C may be avoided by the use of H_3_PO_4_ as the ionomer at concentrations above 105%, which would suppress undesired dehydration of the ionomer, although there is a possibility that the ohmic and polarization resistances of the capacitor would be increased. Alternatively, the functional groups that are introduced onto the carbon surface by HNO_3_ treatment could also contribute to the thermal instability of the capacitor^30^. However, similar instability was observed for the untreated carbon electrode (data not shown); therefore, this effect may be excluded in the present case.

In summary, a SAPO-PTFE composite electrolyte, 105% H_3_PO_4_ electrode ionomer, and HNO_3_-activated carbon electrode were prepared and characterized as cell components for a supercapacitor capable of operation at elevated temperatures. The performance of an EDL capacitor fabricated with optimized cell components was measured from room temperature to 200°C. The capacitance of the capacitor charged to 1 V at 200°C was 210 F g^−1^, which is consistent with that expected from the BET surface area of the carbon electrode material. The energy density was considerably influenced by the operating conditions, in that it 1) increased with temperature to *ca.* 30 Wh kg^−1^ at 200°C; 2) remained high at 12 Wh kg^−1^, even at 13 A g^−1^; 3) had different voltage windows, depending on the operation temperature; and 4) remained unchanged or decreased to 83% of the initial energy density after 7000 cycles from room temperature to 150°C, above which it became gradually lower as the cycle number increased.

## Methods

### Materials

SIPO, SAPO, and FTPO electrolyte membranes were synthesized according to a previously reported procedure^14^,^15^,^16^. For example, in the case of SIPO, SnO_2_ and In_2_O_3_ were mixed with 85% H_3_PO_4_ and deionized water. The mixture was stirred at 300°C until a high viscosity paste was formed. The paste was calcined in an alumina pot at 650°C for 2.5 h and then ground into a powder with a mortar and pestle. 0.04 g of PTFE powder was added to 1.00 g of SIPO powder, kneaded using a mortar and pestle, and then cold-rolled to a thickness of 250 μm using a laboratory rolling mill. Commercially available 105% H_3_PO_4_ (Aldrich) was used as the ionomer for the electrode without further purification. The activated carbon (Maxsorb MSC-30) used in this study was purchased from Kansai Coke and Chemicals. This carbon was modified by stirring 0.1 g of carbon in 50 mL of 1 N HNO_3_ at room temperature for 1 h. After filtering, washing, and drying, the weight of the recovered sample was recorded. For comparison, three types of carbon black (Vulcan XC-72R, Ketjen Black EC600JD, Black Pearls 2000) with different surface areas were employed.

### Characterization

The ionic conductivity of the electrolyte or ionomer samples was estimated using an electrochemical cell composed of the sample with Au plate electrodes, where a piece of filter paper was used as a separator only for H_3_PO_4_. EIS was used to analyze the cells with a Solartron SI 1260 impedance analyzer and a Solartron 1287 electrochemical interface in the frequency range of 10–10^6^ Hz with an AC amplitude of 10 mV. TG-DTA curves for the H_3_PO_4_ ionomers were observed using a Shimadzu DTG-60 instrument from room temperature to 300°C in a N_2_ flow at a heating rate of 10°C min^−1^. The morphology and microstructure of the samples was examined using SEM (Keyence, VE-8800) and TEM (Jeol JEM2100F), respectively. The chemical charge states of C 1s and O 1s were analyzed using XPS (VG Escallab220i-XL) with an Al Kα (1486.6 eV) X-ray source. The photoemission angle was set at 45° to the sample surface, allowing for a reduction in the escape depth to a few nanometers. The crystallinity of the samples was quantified using a Raman spectrophotometer (Jasco NRS-1000) with visible (532 nm) laser excitation. The pore characteristics of the samples were measured by N_2_ adsorption at liquid N_2_ temperature and the specific surface areas were calculated using the BET method from adsorption data in the relative pressure range of 0.001 to 0.3. The micro- and mesopore volume distributions were determined using the standard MP and BJH methods, respectively.

### Supercapacitor assembly

The electrode was prepared using the following procedure. A mixture of the carbon powder, 60% PTFE dispersion, 2-propanol, and deionized water was dispersed using a mixer (Thinky AR-100). The slurry was deposited on the surface of a carbon fiber paper (Toray TGP-H-090) using a screen-printing technique and then dried at 120°C in air to remove the solvent. The loading of carbon was adjusted to *ca.* 4.5 mg cm^−2^. Two identical 12 mm diameter electrode disks were punched from the carbon fiber paper. Prior to fabrication of the supercapacitor assembly, these electrodes were immersed in the H_3_PO_4_ ionomer. A 14 mm diameter electrolyte membrane was sandwiched between the two electrodes attached to stainless steel current collectors. The supercapacitor was then sealed using thermal- and chemical-resistant PTFE tape (Nitto Denko, Nitoflon).

### Electrochemical measurements

All electrochemical measurements were conducted by fabricating symmetric capacitors with the two identical electrodes (two-electrode cell). The withstanding voltage properties of each cell component were determined by CV measurements. CV profiles were collected between −2.5 and +2.5 V using a galvanostat/potentiostat (Hokuto Denko HZ-5000) at a scan rate of 10 mV s^−1^. The internal resistance and capacitive behavior of the capacitors were analyzed using EIS measurements. Impedance spectra were acquired in a similar manner to that for the ionic conductivity measurements, except that the minimum frequency was set at 0.1 Hz. The capacitance and energy density of the capacitors were obtained from galvanostatic charge-discharge measurements. The voltage during charge and discharge was monitored using an impedance analyzer (Solartron SI 1260). The capacitance, energy density, and current density were normalized according to the total mass (*ca.* 10 mg) of the two carbon electrodes.

## Author Contributions

T.H. proposed the original concept and wrote the manuscript. K.K. and M.N. performed research. T.H. and S.K. discussed the experimental results.

## Supplementary Material

Supplementary Information Supplementary Information

## Figures and Tables

**Table 1:** Physicochemical properties of non-treated and HNO_3_-treated Maxsorb

Sample	Weight/g	O/C (XPS)	D/G (Raman)	BET surface area/m^2^ g^−1^
Non-treated				
Maxsorb	1.00	0.044	1.00	2955
HNO_3_-treated				
Maxsorb	1.08	0.066	0.96	2594
