# Supplementary material for: High-temperature supercapacitor with a proton-conducting metal pyrophosphate electrolyte
Source: Sci Rep. 2015 Jan 20;5:7903. doi: 10.1038/srep07903 (PMC4298720; doi:10.1038/srep07903)
Supplement: Supplementary Information [file srep07903-s1.pdf]

Supplementary Information

High-temperature supercapacitor with a proton-conducting metal pyrophosphate electrolyte

Takashi Hibino,[1,*] Kazuyo Kobayashi,[1] Masahiro Nagao,[1] and Shinji Kawasaki[2]

[1]Graduate School of Environmental Studies, Nagoya University, Furo-cho, Chikusa-ku, Nagoya 464-8601, Japan

[2]Graduate School of Engineering, Nagoya Institute of Technology, Gokiso-cho, Showa-ku, Nagoya 466-8555, Japan

[*]Corresponding author, E-mail address: hibino@urban.env.nagoya-u.ac.jp, Tel: +81-52-789-4888, Fax: +81-52-789-4894*

Figure S1. He penetration rate through the SIPO composite as a function of membrane thickness.

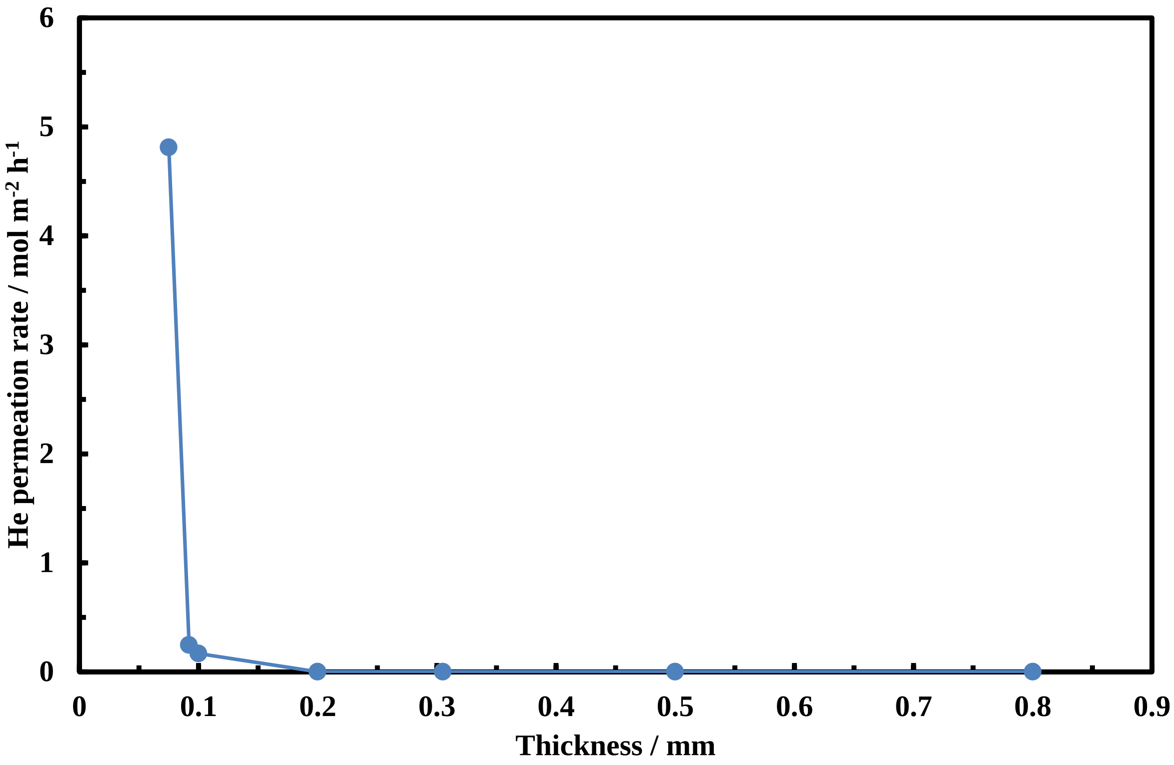

The helium permeability of the membrane sample was determined at 150 °C by preparing two chambers separated by the membrane. Pure helium was supplied to one chamber and argon to the other at a flow rate of 60 mL min^{-1}; the amount of helium that entered the argon chamber was monitored using a gas chromatograph (Shimadzu, GC-8A) with a thermal conductivity detector.

Figure S2. SEM images before and after immersion of the electrode ionomer into 105% H_3PO_4.

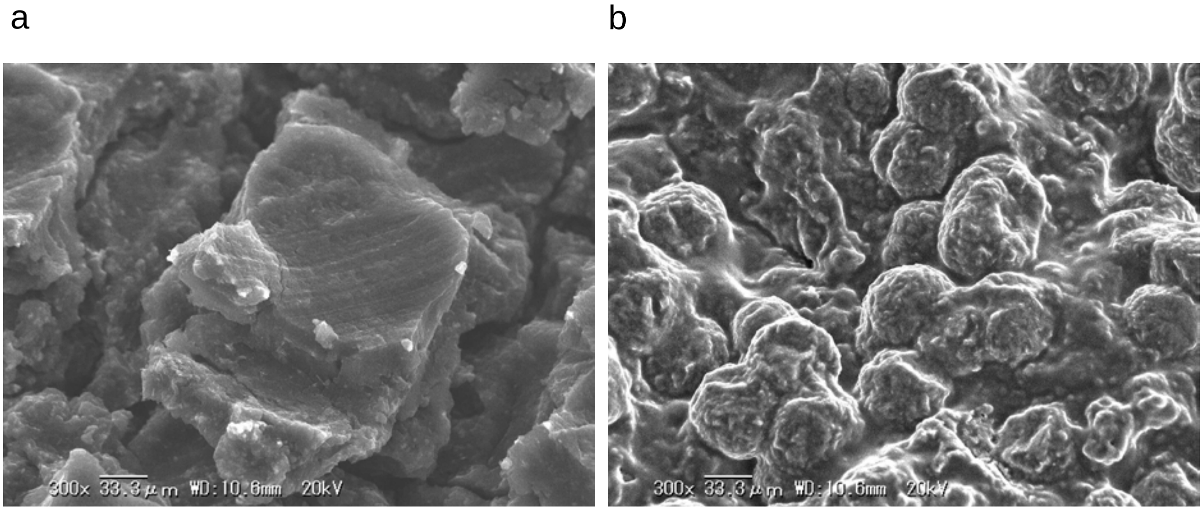

The carbon particles are homogeneously and intimately covered with 105% H_3PO_4, which suggests that the wettability between the two media is good, at least for the external surface of the carbon particles.

Figure S3. Galvanostatic charge-discharge curve measured for pure untreated Maxsorb with a current density of 3.6 A g^{-1} at 200 °C.

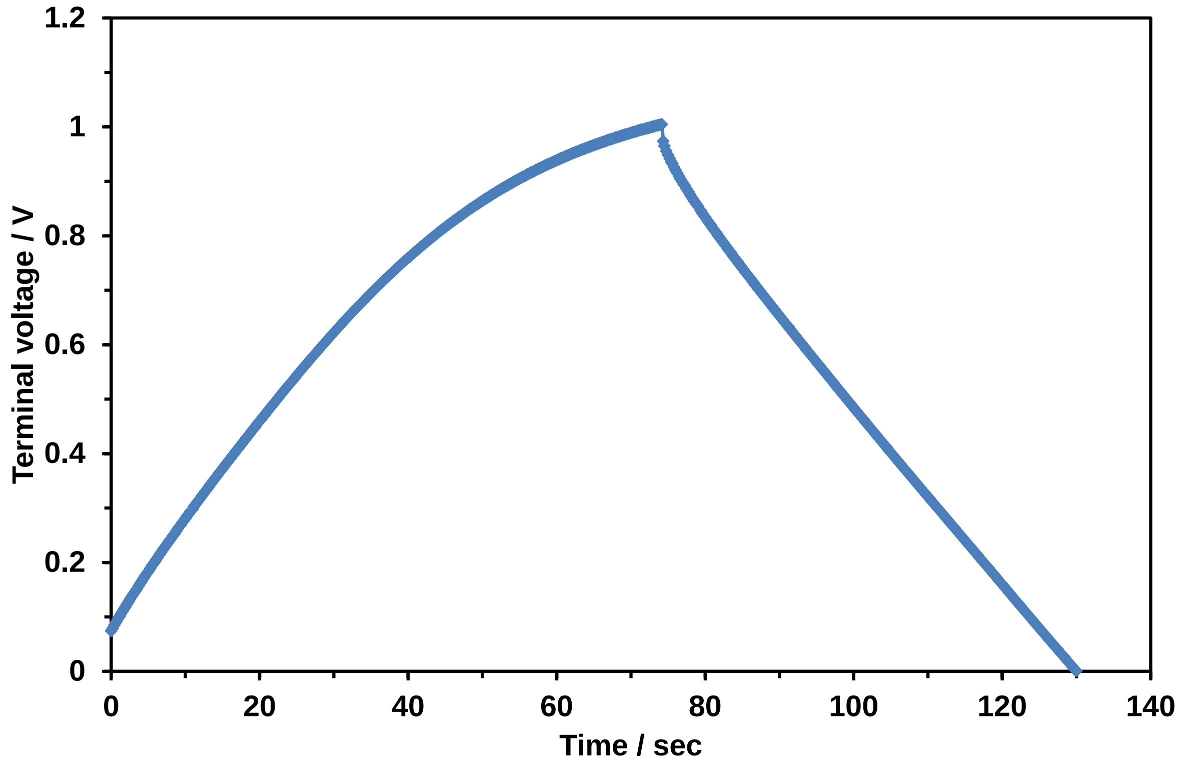

Pure untreated Maxsorb was used as the carbon electrode. The capacitance (C) was calculated according to:

$$C = \frac{I \times t}{\Delta V \times w}$$

where I is the discharge current, t is the discharge time, ΔV is the voltage difference of discharge, and w is the total mass of the two carbon electrodes. The carbon sample showed a capacitance of 173 F g^{-1}.

Figure S4. XPS spectra for the untreated and HNO_3-treated Maxsorb.

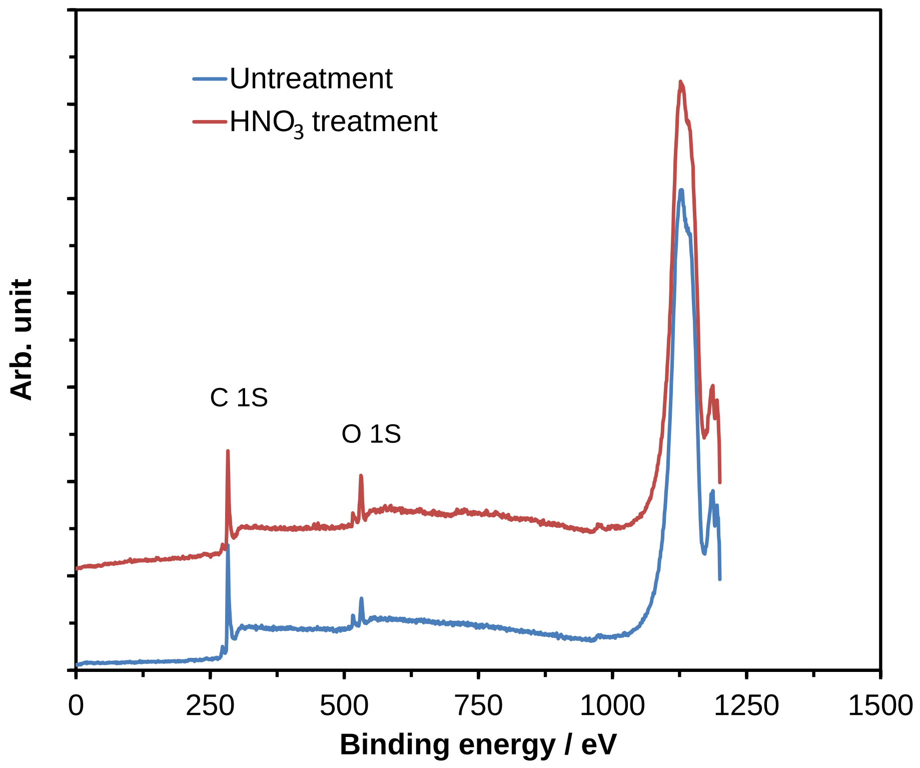

The O/C atomic ratio increased from 0.044 to 0.066 by HNO_3 treatment at room temperature, which is consistent with the weight increase of the carbon sample after modification.

Figure S5. Raman spectra for the untreated and HNO_3-treated Maxsorb.

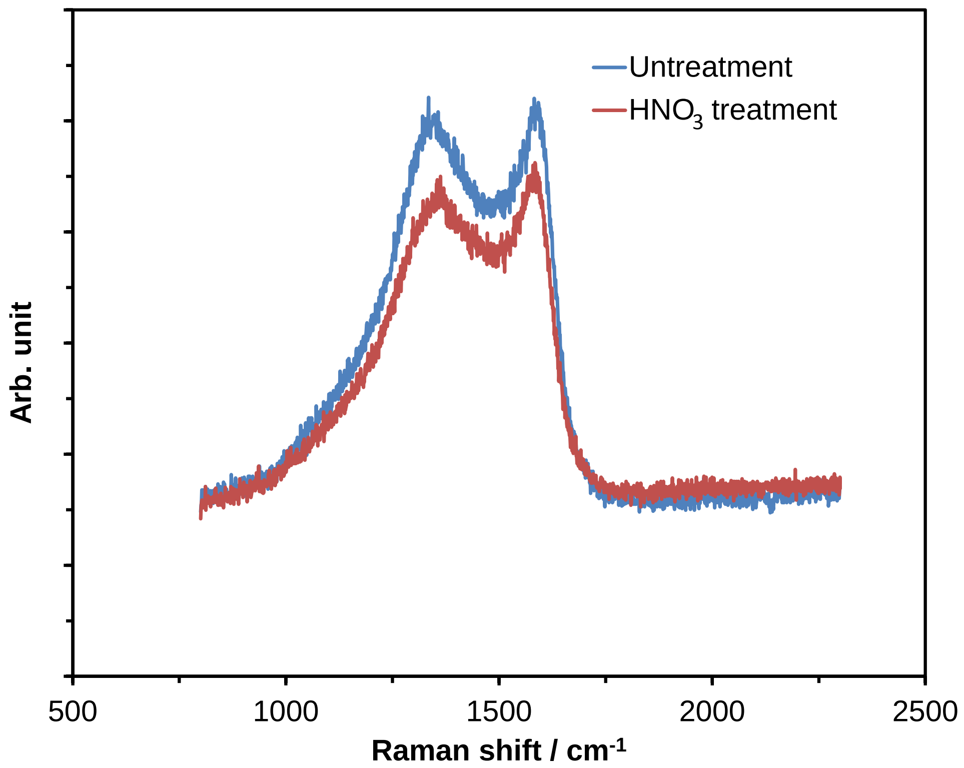

The Raman spectra showed no difference in the intensity ratio of the D-band (*ca*. 1340 cm^{-1}) to the G-band (*ca*. 1580 cm^{-1}) between the two samples, which implies that the carbon sample did not undergo significant structural deformation.

Figure S6. EIS spectra for the capacitor with the SAPO-PTFE composite electrolyte, 105% H_3PO_4 electrode ionomer, and HNO_3-activated carbon measured at various temperatures.

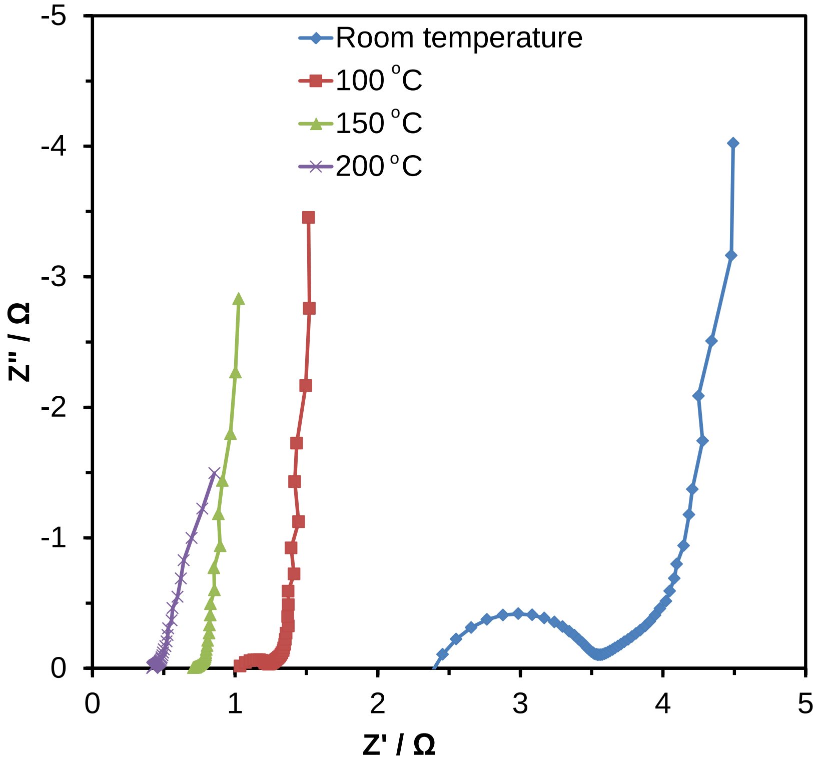

The measured Nyquist plots showed ideal capacitive behavior at low frequencies with near vertical lines parallel to the imaginary axis. However, a slight deviation from the theoretical 90° vertical line was observed for the capacitor at 200 °C, which indicates that the carbon electrode does not function as a planar electrode under such conditions.

Figure S7. Galvanostatic charge-discharge curves for a capacitor with the SAPO-PTFE composite electrolyte, 105% H_3PO_4 electrode ionomer, and HNO_3-activated carbon measured at various voltages, where the current density was set at 4.5 A g^{-1}.

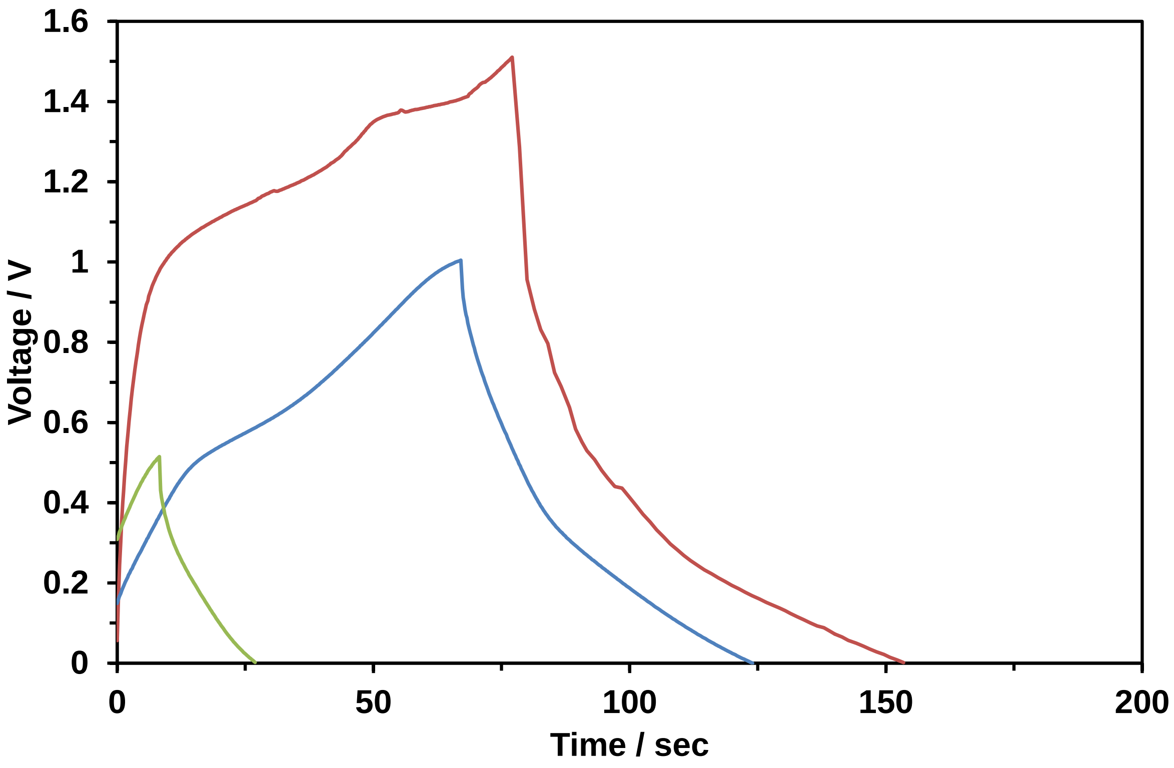

The cell voltage cannot reach 2 V at 4.5 A g^{-1}; therefore, the corresponding data is not shown in this figure. The voltage of the capacitor charged to 1.5 V decreased rapidly from 1.5 to approximately 1 V over time, which suggests that a faradaic reaction proceeds simultaneously with the formation of the EDL during cell charge.
